# IgE-defined endotypes reveal distinct clinical profiles of prurigo nodularis compared with atopic dermatitis: a multicenter study in China

**DOI:** 10.3389/falgy.2026.1769768

**Published:** 2026-02-25

**Authors:** Shihua Ling, Yan Liao, Chaofeng Chen, Ruimiao Wu, Mengting Yin, Lu Wei, Jiaying Chen, Haiyan Huang, Xia Dou

**Affiliations:** 1 Department of Dermatology, Peking University Shenzhen Hospital, Shenzhen, China; 2 Shantou University Medical College, Shantou, China; 3 Shenzhen Key Laboratory for Translational Medicine of Dermatology, Institute of Dermatology, Shenzhen Peking University-The Hong Kong University of Science and Technology Medical Center, Shenzhen, China

**Keywords:** atopic dermatitis, case-control study, China, immunoglobulin E, prurigo nodularis

## Abstract

**Background:**

Prurigo nodularis (PN) is a chronic inflammatory dermatosis that often coexists with atopic dermatitis (AD) but represents a distinct entity. Evidence directly comparing PN and AD in Chinese populations remains limited.

**Objectives:**

To characterize clinical distinctions between PN and AD in Chinese adults and to explore whether total serum immunoglobulin E (IgE) status delineates distinct clinical profiles within PN.

**Methods:**

This multicenter case–control study enrolled 2,621 adult patients (≥18 years) including 1,462 with PN and 1,159 with AD. Multivariable logistic regressions were used to evaluate independent associations differentiating PN from AD and to examine IgE-defined clinical heterogeneity within PN.

**Results:**

Compared with AD, PN was independently associated with middle age, rural residence, lower education, smoking, and an overall reduced atopic profile, and was more frequently accompanied by type 2 diabetes mellitus and psychiatric disorders. Within PN, the IgE-high subgroup displayed a pronounced type 2 immune response-associated inflammatory profile, including peripheral eosinophilia and allergen sensitization, whereas IgE-normal patients showed comparatively attenuated atopic markers. In contrast, sociodemographic correlates that distinguished PN from AD were not consistently different between IgE-defined PN subgroups.

**Conclusions:**

In Chinese adults, PN exhibits clinical heterogeneity with two distinct patterns: one characterized by variability in type 2 immunity-associated and atopic features, and the other by a substantial burden of specific sociodemographic and metabolic comorbidities. Serum IgE may serve as a practical marker for stratifying type 2 immunity and atopic variability in PN.

## 1 Introduction

Prurigo nodularis (PN) is a chronic inflammatory dermatosis characterized by persistent pruritus, repeated scratching, and multiple hyperkeratotic nodules (1). These distressing symptoms severely disrupt sleep and mental health, impair quality of life, and increase healthcare utilization (2). The disease burden of PN is considerable, with a U.S. prevalence of approximately 72 per 100,000 adults aged 18–64 years based on 2016 nationwide claims data and marked variability across European populations (3–5). In Asia, a Korean dermatology outpatient cohort reported increasing PN-related clinic visits and prescriptions from 2007 to 2020 (6).

Real-world evidence consistently indicates that PN frequently coexists with chronic pruritic dermatoses and systemic comorbidities, especially atopic diseases such as atopic dermatitis (AD), allergic rhinitis, and asthma. In addition, cardiometabolic disorders, renal disease, sleep disturbance, and psychiatric conditions are also common (7–9). Among dermatologic comorbidities, AD was prominent in a large UK database analysis (4). Clinically, a subset of patients with AD may develop pruriginous nodules driven by the chronic itch–scratch cycle, presenting as the so-called prurigo phenotype of AD (10). Nevertheless, converging clinical and translational evidence supports PN as an independent disease entity rather than a variant of AD (10, 11). In international comparative studies and authoritative reviews, PN differs markedly from AD, being more prevalent in older adults and associated with more severe itch and greater quality-of-life impairment (1, 2). Mechanistically, studies using single-cell and spatial omics have identified profibrotic fibroblast activation and IL-31–driven itch signaling as key mechanisms that distinguish PN from eczematous AD (12, 13). Reflecting these insights, European S2k and IFSI guidelines recommend an indication-driven, stepwise diagnostic workup to identify atopy and systemic comorbidities. Assessment of total IgE and, when appropriate, allergen-specific IgE is advised when an atopic diathesis is suspected (11, 14).

Despite broadly international observations, direct comparative studies of PN and AD in Chinese adults remain limited. Existing multicenter investigations have primarily described comorbidity profiles and overall disease burden rather than phenotypic distinctions (15). Against this background, the present multicenter case–control study was designed to characterize the distinctive clinical features of PN compared with AD and to explore heterogeneity within PN based on total serum IgE status.

## 2 Materials and methods

### 2.1 Study design and participants

This study was a multicenter retrospective case–control study based on the China Type II Inflammatory Skin Disease Clinical Research and Standardized Diagnosis and Treatment Project (NCT05316805). Adult patients (≥18 years) with PN or AD were recruited from more than 100 hospitals in China between June 2021 and January 2024. To enhance comparability, eligible adult patients with PN and AD were enrolled during the same study period at participating centers using standardized diagnostic criteria and harmonized recruitment and data-collection procedures. PN was diagnosed based on predefined clinical criteria, including chronic pruritus for more than 6 weeks, persistent scratching, and the presence of localized or generalized nodular lesions (16). AD was diagnosed according to the Williams Diagnostic Criteria (17). The study was approved by the Clinical Research Ethics Committee of Peking University First Hospital (Approval No. 2021/223) and the Ethics Committee of Peking University Shenzhen Hospital (Approval No. 2022/022). All participants provided written informed consent at enrollment. Supplementary Figure S1 shows the flowchart of the participant selection process for the study.

### 2.2 Data collection

At enrollment, sociodemographic data, including age, sex, body mass index (BMI), residence, occupation, education, smoking status, and alcohol use were collected. Additionally, allergic and atopic histories, hematologic and allergy-related biomarkers, and comorbidities were recorded. Overall clinical status and characteristics were documented along with standardized assessments by dermatologists, such as the Investigator's Global Assessment (IGA), peak pruritus Numerical Rating Scale (pp-NRS), Dermatology Life Quality Index (DLQI), and Hospital Anxiety and Depression Scale (HADS).

### 2.3 Definition of study groups and covariates

Four prespecified comparisons were defined: (1) PN vs. AD (Reference: AD); (2) IgE-high PN vs. IgE-normal PN (Reference: IgE-normal PN); (3) IgE-high PN vs. AD (Reference: AD); and (4) IgE-normal PN vs. AD (Reference: AD). PN subgroups were defined based on a site-reported binary indicator of elevated total serum IgE at enrollment, using each center's local laboratory reference range. Absolute IgE values and assay-specific thresholds were not available in the analytic dataset. Therefore, IgE status was treated as a pragmatic indicator rather than a standardized cutoff. Covariates included demographic and lifestyle factors, atopic and clinical allergy history, hematologic and allergy-related biomarkers, and comorbidities. Age was analyzed in categories (18–44, 45–59, 60–74, ≥75 years), and BMI was grouped based on World Health Organization standards.

### 2.4 Statistical analysis

Baseline characteristics were summarized using descriptive statistics. Multivariable logistic regression was used to estimate adjusted odds ratios (aORs) with 95% confidence intervals (CIs) for the prespecified comparisons. For each binary comparison, the dependent variable was coded with the target group as 1 and the reference group as 0; therefore, an aOR > 1 indicates higher odds of belonging to the target group relative to the stated reference group. Group ordering and labels for each comparison are provided in the figure and table legends. Missing data were handled using multiple imputation (m = 20). Imputed datasets were analyzed separately and pooled using Rubin's rules. Total IgE status was handled differently across analyses. In the primary PN vs. AD comparison, IgE status was treated as a covariate and was included in the imputation procedure when missing. In contrast, in the within-PN analyses in which PN was stratified by IgE status, IgE status defined subgroup membership and was not imputed, and the stratified analyses were therefore restricted to participants with observed IgE status. Multicollinearity was assessed using generalized variance inflation factors, and statistical significance was defined as a two-sided *P* < 0.05.

To understand how IgE status might alter the clinical profile, the analysis tested for interactions between IgE status and key atopic covariates. Age and BMI were additionally explored with restricted cubic splines in supplementary analyses to visualize nonlinear patterns, while the primary analyses retained categorical specification for ease of clinical interpretation.

Discrimination was evaluated using the area under the receiver operating characteristic (AUC) curve, with internal validation using bootstrapping (1,000 bootstrap samples). Calibration was assessed using calibration intercept and slope and the Brier score. All data processing and analyses were conducted using R statistical software version 4.4.1.

## 3 Results

### 3.1 Participant characteristics

A total of 2,621 participants were enrolled in this study, including 1,462 with PN and 1,159 with AD. The median age was 51.8 years (IQR, 38.1–62.3) for PN, and 42.4 years (IQR, 29.7–60.0) for AD. The proportion of females was similar between the two groups. PN was more prevalent among rural residents and those with lower educational levels, whereas AD was characterized by a higher frequency of atopic features. Disease severity at enrollment was generally moderate in both groups, with IGA=3 being the most frequent category. Compared with AD, PN showed a lower patient-reported symptom burden and less quality-of-life impairment: the median pp-NRS was 6.0 (IQR, 3.0–8.0) in PN and 7.0 (IQR, 5.0–8.0) in AD, and correspondingly, the median DLQI was 9.0 (IQR, 4.0–14.0) and 11.0 (IQR, 6.0–16.0), respectively. The median HADS total score was 10.0 (IQR, 3.0–18.0) for PN and 10.0 (IQR, 3.0–16.0) for AD. Additional details are provided in Table 1. Furthermore, the baseline demographic and clinical characteristics of PN patients with and without a history of AD are presented in Supplementary Table S1.

**Table 1:** Baseline demographic characteristics of atopic dermatitis and prurigo nodularis.

Characteristics	Atopic dermatitis (*n* = 1,159)	Prurigo nodularis (*n* = 1,462)	*P*-value
Age, years, median [Q1, Q3]	42.4 [29.7, 60.0]	51.8 [38.1, 62.3]	<0.001
Age categories, *n* (%)			<0.001
18–44	615 (53.1)	519 (35.5)	
45–59	254 (21.9)	518 (35.4)	
60–74	210 (18.1)	327 (22.4)	
≥75	80 (6.9)	98 (6.7)	
Sex, *n* (%)			0.750
Female	522 (45.0)	648 (44.4)	
Male	637 (55.0)	812 (55.6)	
BMI, median [Q1, Q3]	22.8 [20.7, 24.9]	23.4 [21.3, 25.4]	<0.001
BMI categories, *n* (%)			<0.001
<18.5	90 (7.8)	65 (4.7)	
18.5–24.9	795 (68.8)	911 (66.0)	
25.0–29.9	209 (18.1)	307 (22.2)	
≥30.0	61 (5.3)	98 (7.1)	
Residence, *n* (%)			<0.001
Urban	1,040 (89.9)	1,081 (78.2)	
Rural	117 (10.1)	302 (21.8)	
Education, *n* (%)			<0.001
Primary or below	96 (8.3)	219 (15.8)	
Lower secondary	216 (18.7)	365 (26.4)	
Upper secondary	215 (18.6)	320 (23.1)	
Associate degree	222 (19.2)	244 (17.6)	
Bachelor's or higher	408 (35.3)	236 (17.1)	
Occupation, *n* (%)			<0.001
Employed	115 (9.9)	46 (3.3)	
Student	392 (33.9)	443 (31.9)	
Unemployed	223 (19.3)	331 (23.9)	
Retired	61 (5.3)	73 (5.3)	
Other	366 (31.6)	494 (35.6)	
Lifestyle, *n* (%)			
Smoking	82 (7.2)	166 (12.6)	<0.001
Alcohol	64 (5.6)	93 (7.0)	0.160
Atopy & sensitivities, *n* (%)			
Elevated total serum IgE	313 (27.1)	245 (17.6)	<0.001
Peripheral blood eosinophilia	215 (18.6)	165 (11.8)	<0.001
Allergen-specific IgE (≥ class 2)	145 (12.5)	65 (4.7)	<0.001
Immediate hypersensitivity reactions	45 (3.9)	31 (2.2)	0.019
Food allergy	69 (6.0)	31 (2.3)	<0.001
Drug allergy	21 (1.8)	20 (1.5)	0.530
Family history of atopic diseases	249 (21.5)	138 (9.9)	<0.001
Comorbidities, *n* (%)			
Asthma	64 (5.5)	36 (2.7)	<0.001
Allergic rhinitis	336 (29.0)	159 (11.8)	<0.001
Allergic conjunctivitis	30 (2.6)	5 (0.4)	<0.001
Chronic urticaria	53 (4.6)	52 (3.8)	0.370
Ichthyosis vulgaris	21 (1.8)	6 (0.4)	0.001
Hypertension	54 (4.7)	94 (7.0)	0.017
Coronary heart disease	9 (0.8)	16 (1.2)	0.320
Type 2 diabetes mellitus	19 (1.6)	44 (3.3)	0.010
Psychiatric disorders	6 (0.5)	13 (1.0)	0.250
Disease severity and patient-reported outcomes			
Investigator's global assessment (IGA, 0–4)			<0.001
0 (clear)	18 (1.6)	290 (19.8)	
1 (almost clear)	33 (2.8)	34 (2.3)	
2 (mild)	297 (25.6)	302 (20.7)	
3 (moderate)	609 (52.5)	613 (41.9)	
4 (severe)	202 (17.4)	223 (15.3)	
Peak pruritus numerical rating scale (pp-NRS)	7.0 [5.0, 8.0]	6.0 [3.0, 8.0]	<0.001
Dermatology life quality index (DLQI, 0–30)	11.0 [6.0, 16.0]	9.0 [4.0, 14.0]	<0.001
Hospital anxiety and depression scale (HADS total score, 0–42)	10.0 [3.0, 16.0]	10.0 [3.0, 18.0]	0.5

Data are presented as observed prior to multiple imputation. Continuous variables are shown as median [IQR] and categorical variables as number (%). Denominators vary across variables due to missing data. Missing data were handled by multiple imputation in regression analyses as described in the Methods. *P* values were calculated using the Wilcoxon rank-sum test for continuous variables and Pearson chi-square tests or Fisher exact tests for categorical variables as appropriate. Investigator's Global Assessment (IGA) is an ordinal severity scale ranging from 0 (clear) to 4 (severe). Abbreviations: BMI, body mass index; IgE, immunoglobulin E; IQR, interquartile range.

### 3.2 Comparison between PN and AD patients

**Figure 1:**
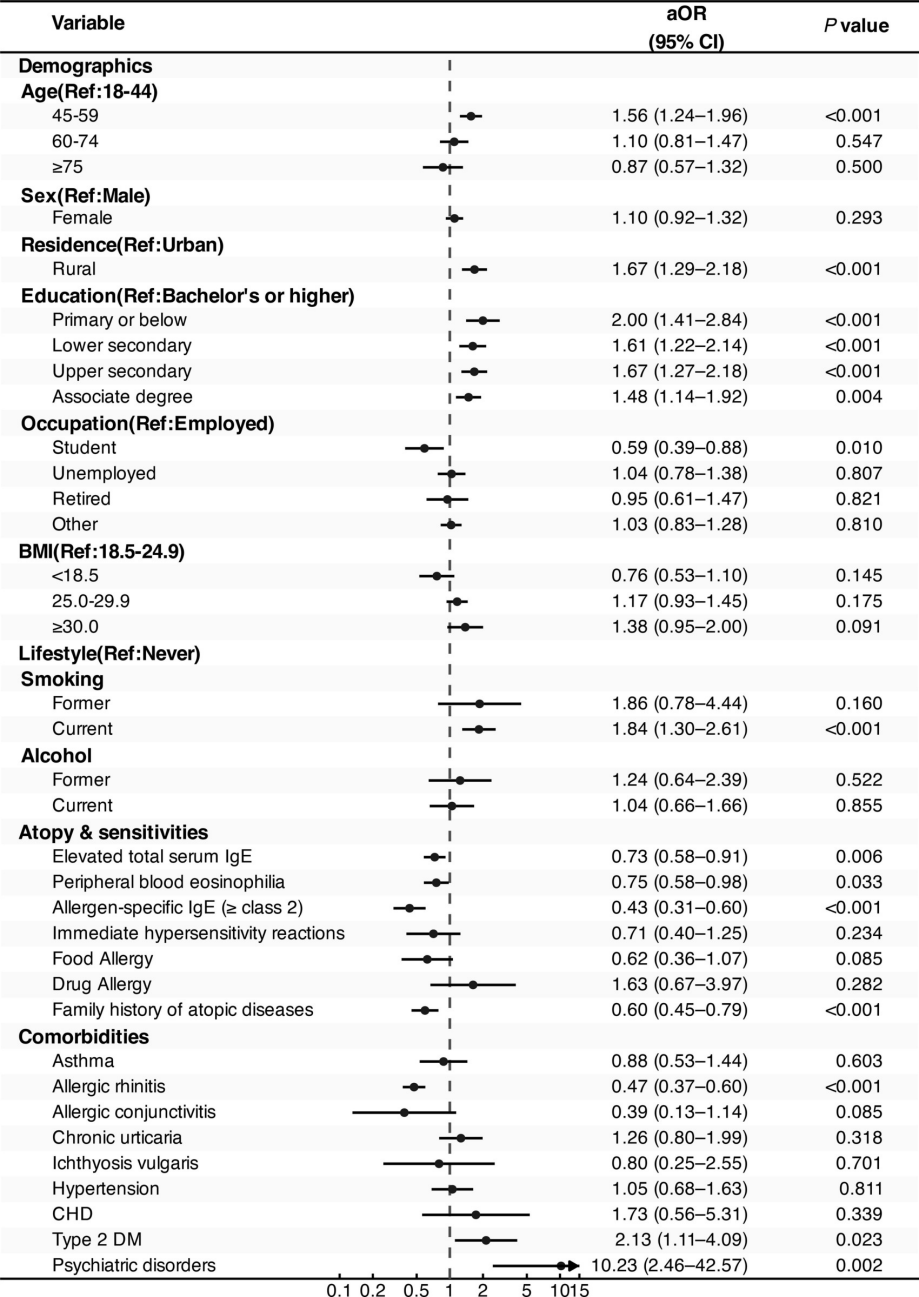
Adjusted associations distinguishing prurigo nodularis (PN) from atopic dermatitis (AD). Adjusted odds ratios (aORs) with 95% confidence intervals (CIs) from multiple-imputation analyses. Atopic dermatitis (AD) served as the reference group for all comparisons. Each point represents the aOR from multivariable logistic regression, with black bars denoting 95% CIs. An aOR > 1 indicates a higher odds of the characteristic for the PN group relative to the AD group. AD, atopic dermatitis; aOR, adjusted odds ratio; CI, confidence interval; PN, prurigo nodularis; IgE, immunoglobulin E; Ref, reference; CHD, coronary heart disease; Type 2 DM, type 2 diabetes mellitus.

Compared with AD, PN was associated with middle age and several socio–behavioral factors. Patients aged 45–59 years (vs. 18–44 years) had higher odds of PN [adjusted odds ratio (aOR), 1.56; 95% CI, 1.24–1.96; *P* < 0.001], as did those residing in rural areas (aOR, 1.67; 95% CI, 1.29–2.18; *P* < 0.001). Lower educational attainment (primary or below vs. bachelor's or higher) was associated with an aOR of 2.00 (95% CI, 1.41–2.84; *P* < 0.001), as was current smoking (aOR 1.84; 95% CI, 1.30–2.61; *P* < 0.001). In contrast, atopic indicators were lower in PN than in AD. These included elevated total serum IgE (aOR 0.73; 95% CI, 0.58–0.91; *P* = 0.006), peripheral eosinophilia (aOR 0.75; 95% CI, 0.58–0.98; *P* = 0.033), allergen-specific IgE class ≥2 (aOR 0.43; 95% CI, 0.31–0.60; *P* < 0.001), the family history of atopic diseases (aOR 0.60; 95% CI, 0.45–0.79; *P* < 0.001), and allergic rhinitis (aOR 0.47; 95% CI, 0.37–0.60; *P* < 0.001). Regarding comorbidities, PN was more frequently associated with type 2 diabetes mellitus (aOR 2.13; 95% CI, 1.11–4.09; *P* = 0.023), and psychiatric disorders were also more frequent (aOR 10.23; 95% CI, 2.46–42.57; *P* = 0.002). However, coronary heart disease and current alcohol use showed no significant associations. These results are presented in Figure 1**.**

**Figure 2:**
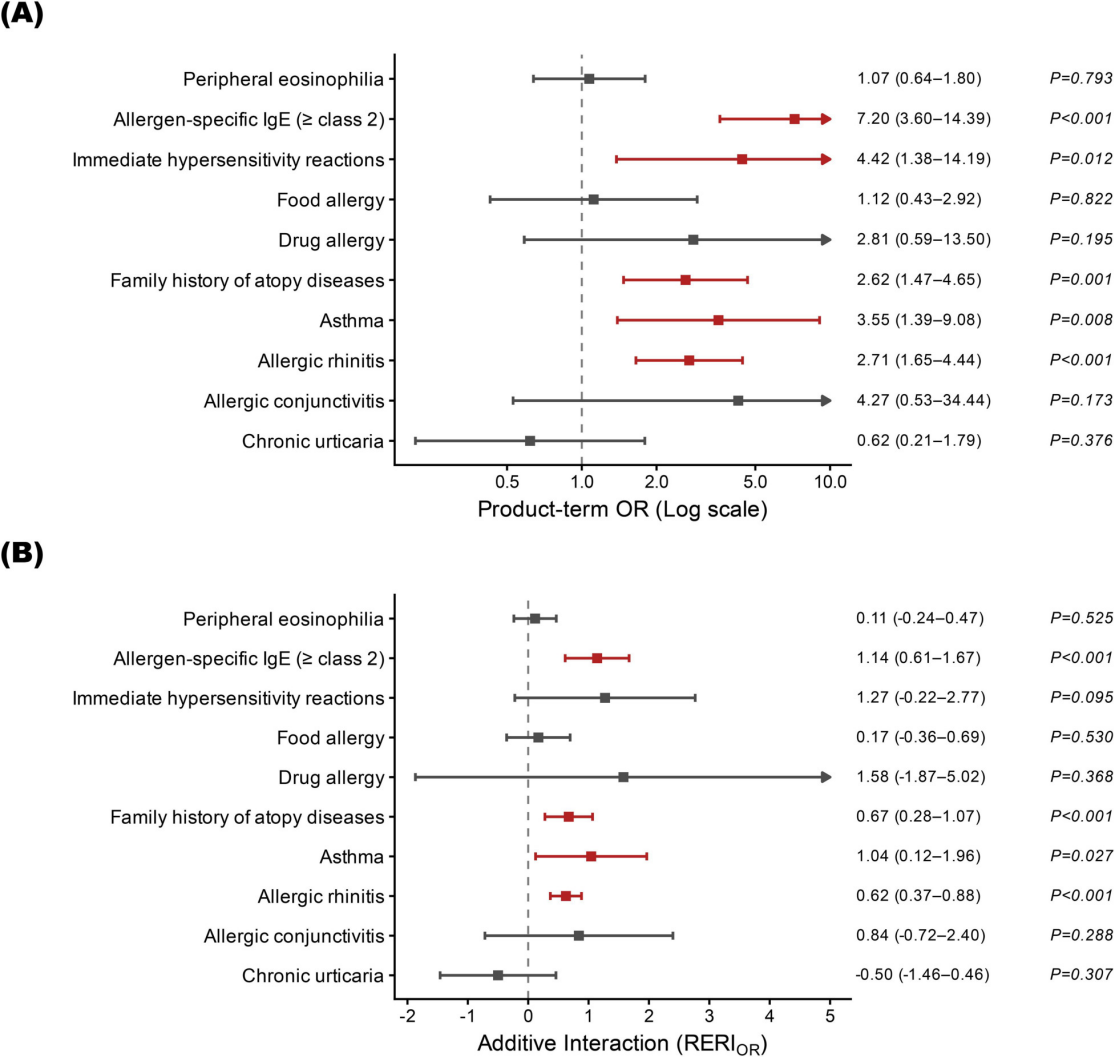
Interaction between Serum IgE and clinical markers in differentiating prurigo nodularis (PN) from atopic dermatitis (AD). **(A)** Multiplicative interaction (product-term odds ratio) with 95% confidence intervals (CIs) on a log scale. **(B)** Additive interaction on the odds scale, expressed as Relative Excess Risk of Interaction (RERI_OR_) with 95% CIs on a linear scale.

Further analyses suggested effect modification by IgE status for atopic features in PN. The attenuation of atopic markers observed for PN overall, exemplified by allergen-specific IgE and allergic rhinitis, appeared to be largely driven by the IgE-normal subgroup, whereas the IgE-high subgroup showed an atopic profile more similar to AD. On the multiplicative scale, tests for interaction between IgE status and classical atopic markers were statistically significant for allergen-specific IgE (*P* for interaction < 0.001), allergic rhinitis (*P* for interaction < 0.001), and asthma (*P* for interaction = 0.008). Figure 2 illustrates these stratified associations, and full interaction estimates are provided in Supplementary Table S2.

**Figure 3:**
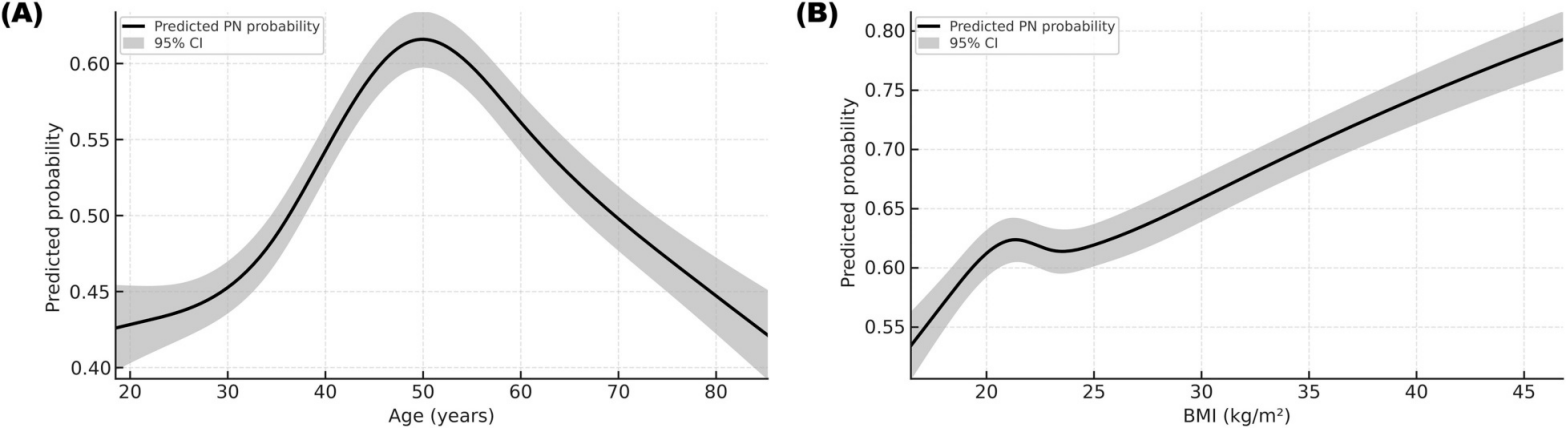
Restricted cubic spline (RCS) partial effects for Age and BMI in PN. **(A)** Adjusted predicted probability across age. **(B)** Adjusted predicted probability across BMI. Curves were generated from logistic models with four knots at the 5th, 35th, 65th, and 95th percentiles (age: 21.35, 39.07, 56.67, 77.19 years; BMI: 18.34, 21.97, 24.22, 31.25 kg/m^2^). Shaded bands represent 95% confidence intervals (CIs). The curves are truncated to the 2.5th–97.5th percentile range for each variable.

The relationships between age and BMI and the odds of PN were found to be nonlinear **(**Figure 3**)**. After adjustment, the odds of PN increased from young adulthood, peaked at approximately 50 years of age, and then attenuated in older patients. For BMI, the odds of PN showed a continuous increase across the observed range (16.5–46.8 kg/m^2^).

### 3.3 Comparison between PN patients by IgE

Among 1,462 patients with PN, total serum IgE were available for 1,395 patients (95.4%). Of these, 245 (17.6%) were classified as IgE-high and 1,150 (82.4%) as IgE-normal (Supplementary Table S3). The 67 cases without IgE data were excluded from the IgE-stratified analyses to maintain internal consistency in subgroup comparisons. To assess the potential impact of excluding these cases, Supplementary Table S4 presents a comparison of the baseline demographic characteristics between the 67 patients who lacked IgE measurements and those with available IgE data.

**Figure 4:**
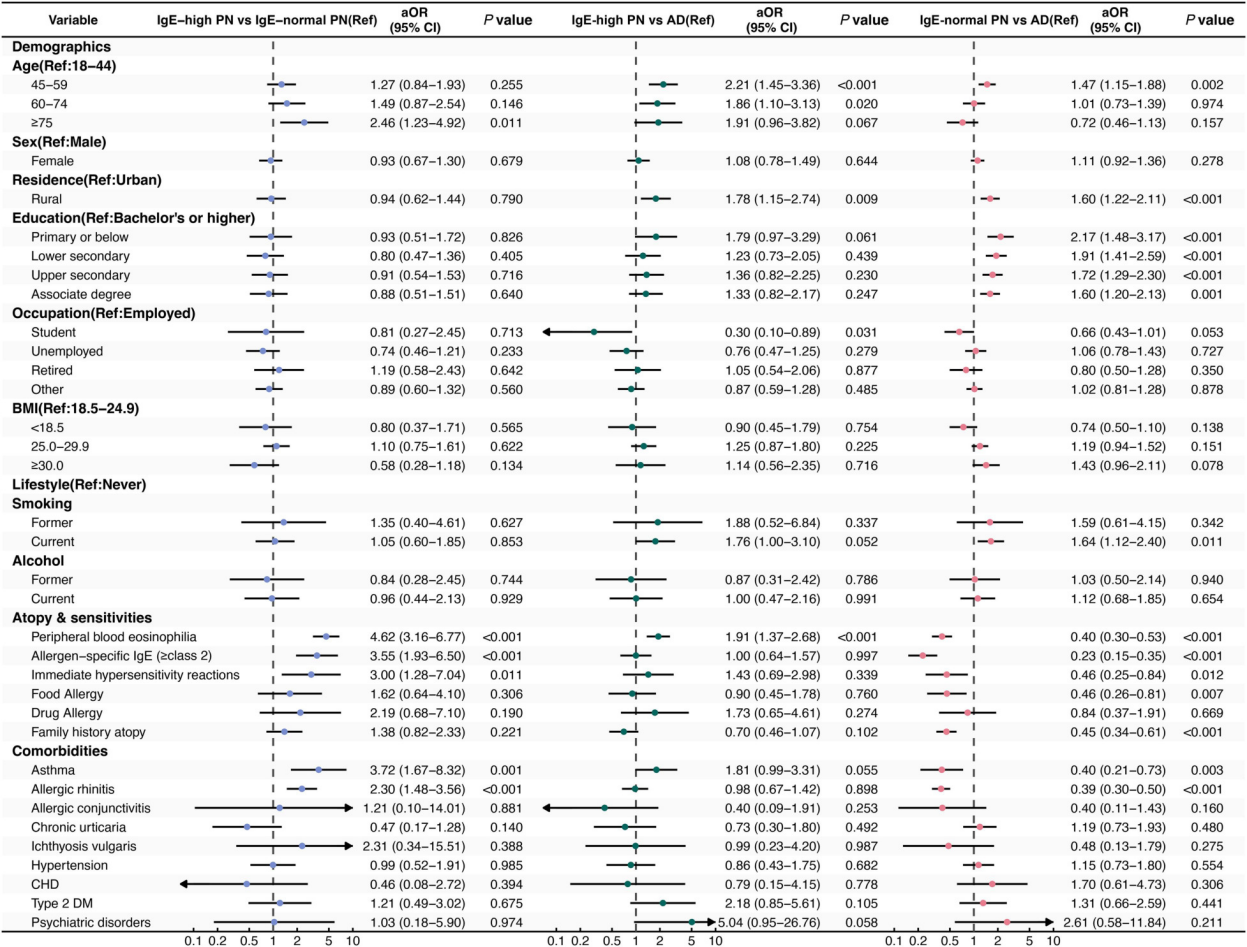
Forest plot of adjusted odds ratios (aORs) with 95% for three prespecified comparisons. (1): Comparison of IgE-high PN to IgE-normal PN (Reference). An aOR > 1 indicates a higher odds of the characteristic for the IgE-high PN subgroup. (2): Comparison of IgE-high PN to AD (Reference). An aOR > 1 indicates a higher odds of the characteristic for the IgE-high PN subgroup. (3): Comparison of IgE-normal PN to AD (Reference). An aOR > 1 indicates a higher odds of the characteristic for the IgE-normal PN subgroup. Each point represents the aOR from multivariable logistic regression, with black bars denoting 95% Confidence Intervals. AD, atopic dermatitis; aOR, adjusted odds ratio; CI, confidence interval; PN, prurigo nodularis; IgE, immunoglobulin E; Ref, reference; CHD, coronary heart disease; Type 2 DM, type 2 diabetes mellitus.

Relative to the IgE-normal PN subgroup, the IgE-high subgroup exhibited a distinctly type 2 immune response-associated inflammatory profile, characterized by peripheral eosinophilia and allergen sensitization. There was a marked increase in peripheral eosinophilia (aOR 4.62; 95% CI, 3.16–6.77; *P* < 0.001) in the IgE-high subgroup, as well as allergen-specific IgE class ≥ 2 (aOR, 3.55; 95% CI, 1.93–6.50; *P* < 0.001). Notably, a key feature defining this atopic endotype was its strong association with advanced age: patients ≥75 years were significantly more likely to be in the IgE-high group (aOR, 2.46; 95% CI, 1.23–4.92; *P* = 0.011). In contrast, there were no significant differences between the subgroups in terms of rural residence and current smoking. These within-PN associations are summarized in Figure 4**.**

### 3.4 Comparison between IgE-high PN and AD patients

When compared to AD, the IgE-high PN subgroup showed limited differences in most type 2 immunity-related biomarkers. Allergen-specific IgE class ≥2 (aOR 1.00; 95% CI, 0.64–1.57; *P* = 0.997), allergic rhinitis (aOR 0.98; 95% CI, 0.67–1.42; *P* = 0.898), and family history of atopic diseases (aOR 0.70; 95% CI, 0.46–1.07; *P* = 0.102) did not differ significantly between groups. However, peripheral eosinophilia was more prevalent in the IgE-high PN patients (aOR 1.91; 95% CI, 1.37–2.68; *P* < 0.001). Sociodemographic correlates characteristic of PN, including age, rural residence, and lower educational attainment, persisted within this IgE-high PN subgroup. Full results are shown in Figure 4**.**

### 3.5 Comparison between IgE-normal PN and AD patients

Relative to AD, the IgE-normal PN subgroup demonstrated a distinct non-atopic profile together with consistent socio–behavioral correlates. Rural residence (aOR 1.60; 95% CI, 1.22–2.11; *P* < 0.001) and lower educational attainment (primary or below; aOR 2.17; 95% CI, 1.48–3.17; *P* < 0.001) were associated with IgE-normal PN. Current smoking was also significantly associated (aOR 1.64; 95% CI, 1.12–2.40; *P* = 0.011). Conversely, atopic features were less prevalent than in AD. Allergen-specific IgE class ≥2 (aOR 0.23; 95% CI, 0.15–0.35; *P* < 0.001) and allergic rhinitis (aOR 0.39; 95% CI, 0.30–0.50; *P* < 0.001) were both significantly less common in the IgE-normal PN subgroup. These stratified associations are illustrated in Figure 4**.**

### 3.6 Model performance and calibration

In the primary analysis distinguishing AD from PN, the model achieved acceptable discrimination (optimism-corrected AUC = 0.713) and reasonable calibration in internal validation. External validation is warranted before applying predicted probabilities to other settings. Full performance metrics for all subgroup analyses are provided in Supplementary Table S5.

## 4 Discussion

This multicenter case–control study provides a descriptive pattern for understanding clinical heterogeneity in PN among Chinese adults. At the disease level, patients with PN differed from those with AD not only in atopy-related features but also in several sociodemographic characteristics, lifestyle factors, and metabolic comorbidities, supporting the concept that PN represents a distinct clinical entity from AD. Within PN, clinical heterogeneity was primarily reflected in serum IgE-related differences. IgE-based stratification identified subgroups that differed mainly in type 2 immunity-associated inflammation and other atopic features. Notably, key sociodemographic factors such as rural residence and smoking, which helped distinguish PN from AD overall, did not significantly differ between the IgE-defined PN subgroups. This suggests that these factors represent a shared clinical background for PN, rather than being linked to a specific immunologic pathway.

Against this backdrop of layered clinical heterogeneity, we examined age-related and comorbidity patterns in PN. In the primary PN vs. AD comparison, PN was more likely to be middle-aged than younger adults, and it remained significantly associated with type 2 diabetes mellitus and psychiatric disorders. This association may reflect a bidirectional vicious cycle: severe chronic itch and sleep disruption exacerbate mental distress, while psychiatric disorders may further lower the itch threshold and drive psychogenic scratching. These findings are consistent with international data indicating a higher disease burden, increased cardiometabolic risk, and greater healthcare utilization in patients with PN (2, 7, 9). Although sex distributions vary across cohorts and healthcare settings (18, 19), the absence of a sex difference in our cohort may reflect specific population sampling or healthcare-seeking patterns.

Our analysis within the PN cohort further indicates that serum IgE serves to characterize immunologic heterogeneity. This interpretation is consistent with prior biomarker-based and proteomic clustering studies that have identified heterogeneous systemic inflammatory signatures and distinct endotypes in PN (20–22). In our cohort, the IgE-high subgroup exhibited clustered eosinophilia and allergen sensitization, presenting an AD-like atopic profile, whereas the IgE-normal subgroup displayed a comparatively attenuated atopic diathesis. Although our subgroups were defined by serum IgE rather than by AD comorbidity, the observed IgE-associated atopic features are in line with earlier clinical descriptions of atopy-associated vs. non–atopy-associated PN and with cohort data highlighting a high prevalence of atopic predisposition among PN patients (23, 24).

An unexpected and hypothesis-generating finding in our cohort was a notable association between advanced age (≥75 years) and the IgE-high PN subgroup. This pattern contrasts with epidemiologic observations indicating that both the prevalence of IgE sensitization and mean total serum IgE tend to decline with advancing age, although allergen sensitization can remain clinically relevant in a subset of older adults (25, 26). Accordingly, the concurrence of an IgE-high classification with peripheral eosinophilia in older patients raises the possibility that sustained type 2 immunity-skewed inflammatory state may characterize a subset of PN in later life. A relevant parallel has been described in asthma, where adult-onset or late-onset eosinophilic phenotypes are consistently recognized within type 2 immunity–high disease patterns, and these phenotypes can exhibit prominent eosinophilic inflammation even when classical allergic sensitization is not a defining feature (27, 28). Taken together, our findings suggest that a comparable geriatric type 2 immunity-skewed phenotype may exist in older patients with PN, which warrants confirmation in prospective studies and mechanistic investigations.

Recent phase 3 clinical trials have demonstrated that IL-4/IL-13 pathway blockade and IL-31 receptor blockade can significantly improve pruritus and nodule severity in PN (29–32). In addition, a small retrospective real-world cohort reported that elevated baseline IgE was associated with greater improvement in pruritus and Investigator's Global Assessment scores among PN patients treated with dupilumab (33). However, as this study was not designed to evaluate treatment response, the IgE-based subgroups should not be considered predictive of treatment outcomes. Therefore, the following treatment considerations are based on established evidence and guidelines, not on our observational data. For patients with PN who exhibit type 2 immunity-associated features, biologic therapies such as IL-4/IL-13 or IL-31 receptor blockade may be considered in accordance with approved indications and current guidelines, alongside optimized topical therapy and phototherapy when appropriate. Supportive management addressing scratching behavior, sleep disturbance, and relevant lifestyle and metabolic factors may also be considered as part of comprehensive care for chronic pruritic disorders (11). Across both IgE-defined subgroups, screening for cardiometabolic disease and psychological burden may be beneficial, because these comorbidities are closely linked to poor itch control and impaired quality of life.

Several limitations should be acknowledged. Residual confounding is possible due to the retrospective multicenter design. IgE testing methods and sensitization thresholds varied across centers, potentially affecting the stratification. Additionally, IgE status was recorded as a site-reported binary indicator, which may have led to misclassification. Patients without IgE data were excluded from the IgE-stratified analysis, which could introduce selection bias. Therefore, IgE-based phenotyping should be interpreted as hypothesis-generating and requires prospective validation using standardized laboratory criteria and improved methods for handling missing data.

In conclusion, this multicenter case–control study demonstrates that PN in Chinese adults exhibits multi-dimensional clinical heterogeneity. Compared with AD, PN showed distinct sociodemographic, lifestyle, and metabolic comorbidity profiles in addition to a relatively attenuated atopic signature, supporting PN as a separate clinical entity. Within PN, serum IgE primarily captured variability in type 2 immunity-related inflammatory biomarkers and other atopic features, whereas comorbidity patterns were broadly similar across IgE-defined subgroups. These findings support the use of IgE as a pragmatic stratification marker for hypothesis-generating phenotyping in PN, but prospective validation with standardized laboratory criteria is needed.

## Data Availability

The raw data supporting the conclusions of this article will be made available by the authors, without undue reservation.

## Supplementary material

Supplementary Figure S1 The flowchart of the participant selection process for the study.

Supplementary Table S1 Baseline characteristics of patients with prurigo nodularis stratified by atopic dermatitis overlap.

Supplementary Table S2 Interaction between IgE and clinical markers.

Supplementary Table S3 Baseline demographic characteristics of prurigo nodularis by IgE subtype.

Supplementary Table S4 Baseline characteristics of patients with prurigo nodularis stratified by availability of total serum IgE status.

Supplementary Table S5 Model performance and internal validation.
