# Supplementary figures and images for: IgE-defined endotypes reveal distinct clinical profiles of prurigo nodularis compared with atopic dermatitis: a multicenter study in China
Source: Front Allergy. 2026 Feb 25;7:1769768. doi: 10.3389/falgy.2026.1769768 (PMC12975732; doi:10.3389/falgy.2026.1769768)

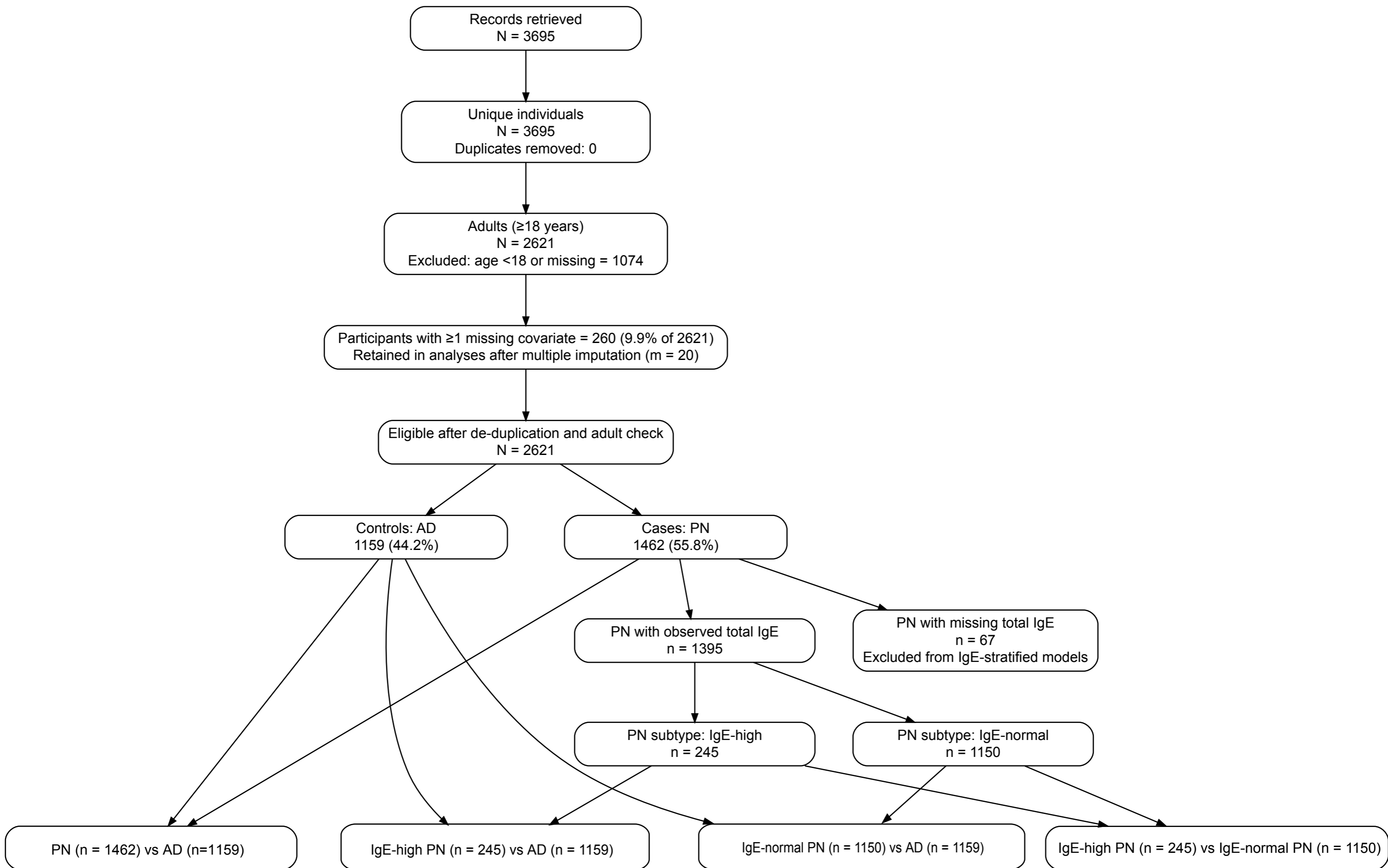

Supplement: Supplementary Figure S1 — The flowchart of the participant selection process for the study. [file Image1.pdf]
