# Supplementary material for: IgE-defined endotypes reveal distinct clinical profiles of prurigo nodularis compared with atopic dermatitis: a multicenter study in China
Source: Front Allergy. 2026 Feb 25;7:1769768. doi: 10.3389/falgy.2026.1769768 (PMC12975732; doi:10.3389/falgy.2026.1769768)
Supplement: Supplementary Table S1 — Baseline characteristics of patients with prurigo nodularis stratified by atopic dermatitis overlap. [file Table1.docx]

**Table S1. Baseline characteristics of patients with prurigo nodularis stratified by atopic dermatitis overlap.**

| Characteristics | PN without AD overlap (n=1157) | PN with AD overlap (n=305) | P value |
| --- | --- | --- | --- |
| **Age, years, median [Q1, Q3]** | 51.4 [37.4, 61.0] | 54.6 [39.7, 66.1] | 0.010 |
| **Age categories, n (%)** |  |  | 0.117 |
| 18–44 | 425 (36.7) | 94 (30.8) |  |
| 45–59 | 409 (35.4) | 109 (35.7) |  |
| 60–74 | 252 (21.8) | 75 (24.6) |  |
| ≥75 | 71 (6.1) | 27 (8.9) |  |
| **Sex, n (%)** |  |  | 0.003 |
| Female | 536 (46.4) | 112 (36.8) |  |
| Male | 620 (53.6) | 192 (63.2) |  |
| **BMI, median [Q1, Q3]** | 23.4 [21.4, 25.5] | 23.4 [21.3, 25.2] | 0.439 |
| **BMI categories, n (%)** |  |  | 0.485 |
| <18.5 | 51 (4.7) | 14 (4.8) |  |
| 18.5-24.9 | 711 (65.3) | 200 (68.5) |  |
| 25.0-29.9 | 244 (22.4) | 63 (21.6) |  |
| ≥30.0 | 83 (7.6) | 15 (5.1) |  |
| **Residence, n (%)** |  |  | 0.039 |
| Urban | 838 (77.0) | 243 (82.7) |  |
| Rural | 251 (23.0) | 51 (17.3) |  |
| **Education, n (%)** |  |  | 0.570 |
| Primary or below | 174 (16.0) | 45 (15.3) |  |
| Lower secondary | 284 (26.1) | 81 (27.6) |  |
| Upper secondary | 259 (23.8) | 61 (20.7) |  |
| Associate degree | 195 (17.9) | 49 (16.7) |  |
| Bachelor's or higher | 178 (16.3) | 58 (19.7) |  |
| **Occupation, n (%)** |  |  | <0.001 |
| Employed | 338 (30.9) | 105 (35.7) |  |
| Other | 410 (37.5) | 84 (28.6) |  |
| Retired | 48 (4.4) | 25 (8.5) |  |
| Student | 43 (3.9) | 3 (1.0) |  |
| Unemployed | 254 (23.2) | 77 (26.2) |  |
| **Lifestyle, n (%)** |  |  |  |
| Smoking, n (%) | 140 (13.5) | 26 (9.2) | 0.055 |
| Alcohol, n (%) | 82 (7.9) | 11 (3.9) | 0.018 |
| **Atopy & sensitivities, n (%)** |  |  |  |
| Elevated total serum IgE | 146 (13.3) | 99 (33.1) | <0.001 |
| Peripheral blood eosinophilia | 96 (8.8) | 69 (23.1) | <0.001 |
| Allergen-specific IgE (≥ class 2) | 36 (3.3) | 29 (9.7) | <0.001 |
| Immediate hypersensitivity reactions | 12 (1.1) | 19 (6.4) | <0.001 |
| Food allergy | 21 (2.0) | 10 (3.5) | 0.179 |
| Drug allergy | 15 (1.4) | 5 (1.7) | 0.593 |
| Family history of atopic diseases | 93 (8.5) | 45 (15.0) | 0.001 |
| **Comorbidities, n (%)** |  |  |  |
| Asthma | 22 (2.1) | 14 (4.9) | 0.013 |
| Allergic rhinitis | 103 (9.7) | 56 (19.5) | <0.001 |
| Allergic conjunctivitis | 4 (0.4) | 1 (0.3) | >0.999 |
| Chronic urticaria | 44 (4.1) | 8 (2.8) | 0.387 |
| Ichthyosis vulgaris | 6 (0.6) | 0 (0.0) | 0.352 |
| Hypertension | 74 (7.0) | 20 (7.0) | >0.999 |
| Coronary heart disease | 13 (1.2) | 3 (1.0) | >0.999 |
| Type 2 diabetes mellitus | 34 (3.2) | 10 (3.5) | 0.851 |
| Psychiatric disorders | 11 (1.0) | 2 (0.7) | >0.999 |
| Data are presented as observed prior to multiple imputation. Continuous variables are shown as median [IQR] and categorical variables as number (%). This table includes PN participants only (n = 1462). Denominators vary across variables due to missing data. Missing data were handled by multiple imputation in regression analyses as described in the Methods. P values were calculated using the Wilcoxon rank sum test for continuous variables and Pearson chi-square tests or Fisher exact tests for categorical variables as appropriate. Note: “PN with AD overlap” indicates prurigo nodularis (PN) participants with concomitant or historical atopic dermatitis (AD) as captured by the study case report form. Abbreviations: PN, prurigo nodularis; AD, atopic dermatitis; IgE, immunoglobulin E; BMI, body mass index. | | | |
