# Supplementary material for: IgE-defined endotypes reveal distinct clinical profiles of prurigo nodularis compared with atopic dermatitis: a multicenter study in China
Source: Front Allergy. 2026 Feb 25;7:1769768. doi: 10.3389/falgy.2026.1769768 (PMC12975732; doi:10.3389/falgy.2026.1769768)
Supplement: Supplementary Table S2 — Interaction between IgE and clinical markers. [file Table2.docx]

## **Table S2. Interaction between IgE and clinical markers**

| Marker | OR10 (IgE+ / marker−) | OR01 (IgE− / marker+) | OR11 (IgE+ / marker+) | Product-term OR (95% CI) | P (multiplicative) | RERI_OR_ (95% CI) | P (RERI) | AP (95% CI) | P (AP) |
| --- | --- | --- | --- | --- | --- | --- | --- | --- | --- |
| Peripheral eosinophilia | 0.72 (0.55–0.93) | 0.73 (0.52–1.03) | 0.56 (0.39–0.82) | 1.07 (0.64–1.80) | 0.793 | 0.11 (-0.24–0.47) | 0.525 | 0.20 (-0.40–0.81) | 0.513 |
| Allergen-specific IgE (≥ class 2) | 0.61 (0.48–0.77) | 0.22 (0.14–0.34) | 0.97 (0.55–1.71) | 7.20 (3.60–14.39) | <0.001 | 1.14 (0.61–1.67) | <0.001 | 1.18 (0.95–1.40) | <0.001 |
| Immediate hypersensitivity reactions | 0.69 (0.55–0.87) | 0.45 (0.24–0.84) | 1.37 (0.49–3.84) | 4.42 (1.38–14.19) | 0.012 | 1.27 (-0.22–2.77) | 0.095 | 0.90 (0.63–1.16) | <0.001 |
| Food allergy | 0.72 (0.57–0.91) | 0.59 (0.32–1.11) | 0.48 (0.21–1.09) | 1.12 (0.43–2.92) | 0.822 | 0.17 (-0.36–0.69) | 0.530 | 0.33 (-0.63–1.29) | 0.497 |
| Drug allergy | 0.71 (0.57–0.89) | 1.10 (0.43–2.82) | 2.20 (0.51–9.45) | 2.81 (0.59–13.50) | 0.195 | 1.58 (-1.87–5.02) | 0.368 | 0.60 (-0.13–1.34) | 0.108 |
| Family history of atopy diseases | 0.62 (0.49–0.79) | 0.46 (0.34–0.63) | 0.75 (0.44–1.26) | 2.62 (1.47–4.65) | 0.001 | 0.67 (0.28–1.07) | <0.001 | 0.89 (0.60–1.18) | <0.001 |
| Asthma | 0.68 (0.54–0.85) | 0.51 (0.27–0.96) | 1.21 (0.58–2.52) | 3.55 (1.39–9.08) | 0.008 | 1.04 (0.12–1.96) | 0.027 | 0.85 (0.53–1.16) | <0.001 |
| Allergic rhinitis | 0.58 (0.45–0.75) | 0.36 (0.27–0.47) | 0.57 (0.37–0.87) | 2.71 (1.65–4.44) | <0.001 | 0.62 (0.37–0.88) | <0.001 | 1.10 (0.76–1.45) | <0.001 |
| Allergic conjunctivitis | 0.71 (0.57–0.90) | 0.24 (0.06–0.89) | 0.73 (0.13–4.19) | 4.27 (0.53–34.44) | 0.173 | 0.84 (-0.72–2.40) | 0.288 | 1.06 (0.35–1.77) | 0.003 |
| Chronic urticaria | 0.74 (0.59–0.94) | 1.43 (0.86–2.39) | 0.66 (0.25–1.73) | 0.62 (0.21–1.79) | 0.376 | -0.50 (-1.46–0.46) | 0.307 | -0.83 (-2.95–1.29) | 0.440 |
| Product-term odds ratios (ORs) with 95% confidence intervals (CIs) describe how high total serum IgE and each marker act together on a multiplicative scale; P (multiplicative) indicates the strength of evidence for such interaction. RERI_OR_ and the attributable proportion (AP) with 95% CIs describe additive interaction, that is, whether high IgE and the marker together increase the odds of prurigo nodularis more than expected from adding their separate effects; P (RERI) and P (AP) indicate the strength of evidence for these excess joint effects. Positive RERI_OR_ and AP values suggest that high IgE and the marker tend to reinforce each other. OR10, IgE-high only; OR01, marker-positive only; OR11, both positive; reference category, IgE-normal and marker-negative. | | | | | | | | | |
