# Supplementary material for: IgE-defined endotypes reveal distinct clinical profiles of prurigo nodularis compared with atopic dermatitis: a multicenter study in China
Source: Front Allergy. 2026 Feb 25;7:1769768. doi: 10.3389/falgy.2026.1769768 (PMC12975732; doi:10.3389/falgy.2026.1769768)
Supplement: Supplementary Table S3 [file Table3.docx]

**Table S3. Baseline demographic characteristics of Prurigo Nodularis by IgE subtype**

| Characteristics | IgE-normal PN (n=1150) | IgE-high PN (n=245) | P value |
| --- | --- | --- | --- |
| **Age, years, Median [Q1, Q3]** | 50.9 [36.7, 60.7] | 55.0 [43.3, 65.8] | <0.001 |
| **Age categories, n (%)** |  |  | 0.005 |
| 18–44 | 433 (37.7) | 68 (27.8) |  |
| 45–59 | 407 (35.4) | 89 (36.3) |  |
| 60–74 | 239 (20.8) | 63 (25.7) |  |
| ≥75 | 71 (6.2) | 25 (10.2) |  |
| **Sex, n (%)** |  |  | 0.228 |
| Female | 521 (45.3) | 100 (41.0) |  |
| Male | 628 (54.7) | 144 (59.0) |  |
| **BMI, Median [Q1, Q3]** | 23.4 [21.3, 25.4] | 23.4 [21.5, 25.6] | 0.707 |
| **BMI categories, n (%)** |  |  | 0.326 |
| <18.5 | 53 (4.7) | 11 (4.8) |  |
| 18.5-24.9 | 742 (66.3) | 151 (65.4) |  |
| 25.0-29.9 | 239 (21.4) | 58 (25.1) |  |
| ≥30.0 | 85 (7.6) | 11 (4.8) |  |
| **Residence, n (%)** |  |  | 0.542 |
| Urban | 872 (77.7) | 184 (79.7) |  |
| Rural | 250 (22.3) | 47 (20.3) |  |
| **Education, n (%)** |  |  | 0.702 |
| Primary or below | 176 (15.7) | 40 (17.3) |  |
| Lower secondary | 305 (27.2) | 54 (23.4) |  |
| Upper secondary | 258 (23.0) | 52 (22.5) |  |
| Associate degree | 199 (17.7) | 41 (17.7) |  |
| Bachelor's or higher | 184 (16.4) | 44 (19.0) |  |
| **Occupation, n (%)** |  |  | 0.044 |
| Employed | 357 (31.7) | 80 (34.6) |  |
| Student | 42 (3.7) | 4 (1.7) |  |
| Unemployed | 263 (23.4) | 58 (25.1) |  |
| Retired | 52 (4.6) | 19 (8.2) |  |
| Other | 411 (36.5) | 70 (30.3) |  |
| **Lifestyle, n (%)** |  |  |  |
| Smoking | 129 (11.9) | 32 (14.5) | 0.311 |
| Alcohol | 72 (6.6) | 18 (8.2) | 0.384 |
| **Atopy & sensitivities, n (%)** |  |  |  |
| Food allergy | 19 (1.7) | 12 (5.3) | <0.001 |
| Drug allergy | 12 (1.1) | 7 (3.1) | 0.028 |
| Family history of atopic diseases | 97 (8.4) | 40 (16.3) | <0.001 |
| Immediate hypersensitivity reactions | 19 (1.7) | 12 (4.9) | 0.002 |
| Peripheral blood eosinophilia | 89 (7.7) | 76 (31.0) | <0.001 |
| Allergen-specific IgE (≥ class 2) | 34 (3.0) | 31 (12.7) | <0.001 |
| **Comorbidities, n (%)** |  |  |  |
| Asthma | 17 (1.5) | 18 (8.0) | <0.001 |
| Ichthyosis vulgaris | 3 (0.3) | 3 (1.3) | 0.063 |
| Allergic rhinitis | 106 (9.5) | 52 (23.1) | <0.001 |
| Allergic conjunctivitis | 3 (0.3) | 2 (0.9) | 0.199 |
| Chronic urticaria | 43 (3.9) | 7 (3.1) | 0.589 |
| Hypertension | 74 (6.6) | 19 (8.4) | 0.332 |
| Coronary heart disease | 14 (1.3) | 2 (0.9) | >0.999 |
| Type 2 diabetes mellitus | 31 (2.8) | 11 (4.9) | 0.098 |
| Psychiatric disorders | 8 (0.7%) | 4 (1.8%) | 0.127 |
| Disease severity and patient-reported outcomes |  |  |  |
| Investigator’s Global Assessment (IGA, 0–4) |  |  | <0.001 |
| 0 (clear) | 226.0 (19.7) | 27.0 (11.0) |  |
| 1 (almost clear) | 18.0 (1.6) | 14.0 (5.7) |  |
| 2 (mild) | 265.0 (23.0) | 33.0 (13.5) |  |
| 3 (moderate) | 481.0 (41.8) | 116.0 (47.3) |  |
| 4 (severe) | 160.0 (13.9) | 55.0 (22.4) |  |
| Peak pruritus Numerical Rating Scale (pp-NRS) | 6.0 [3.0, 8.0] | 7.0 [4.5, 9.0] | 0.014 |
| Dermatology Life Quality Index (DLQI, 0–30) | 9.0 [5.0, 14.0] | 10.0 [4.0, 17.0] | 0.10 |
| Hospital Anxiety and Depression Scale (HADS total score, 0–42) | 10.0 [3.0, 17.0] | 13.0 [2.0, 20.0] | 0.021 |
| Data are presented as observed prior to multiple imputation. Continuous variables are shown as median [IQR] and categorical variables as number (%). This table includes PN participants only (n = 1462). Denominators vary across variables due to missing data. Missing data were handled by multiple imputation in regression analyses as described in the Methods. P values were calculated using the Wilcoxon rank sum test for continuous variables and Pearson chi-square tests or Fisher exact tests for categorical variables as appropriate. Note: Total serum IgE subtype was defined using the site-entered categorical status in the original multicenter database. Abbreviations: AD, atopic dermatitis; PN, prurigo nodularis; IgE, immunoglobulin E; BMI, body mass index; IQR, interquartile range. | | | |
