# Supplementary material for: IgE-defined endotypes reveal distinct clinical profiles of prurigo nodularis compared with atopic dermatitis: a multicenter study in China
Source: Front Allergy. 2026 Feb 25;7:1769768. doi: 10.3389/falgy.2026.1769768 (PMC12975732; doi:10.3389/falgy.2026.1769768)
Supplement: Supplementary Table S4 — Baseline characteristics of patients with prurigo nodularis stratified by availability of total serum IgE status. [file Table4.docx]

**Table S4. Baseline characteristics of patients with prurigo nodularis stratified by availability of total serum IgE status.**

| Characteristics | Total serum IgE measured (n=1395) | Total serum IgE missing (n=67) | P value |
| --- | --- | --- | --- |
| **Age, years, median [Q1, Q3]** | 51.5 [37.7, 62.1] | 57.3 [42.7, 64.7] | 0.050 |
| **Age categories, n (%)** |  |  | 0.019 |
| 18–44 | 501 (35.9) | 18 (26.9) |  |
| 45–59 | 496 (35.6) | 22 (32.8) |  |
| 60–74 | 302 (21.6) | 25 (37.3) |  |
| ≥75 | 96 (6.9) | 2 (3.0) |  |
| **Sex, n (%)** |  |  | 0.531 |
| Female | 621 (44.6) | 27 (40.3) |  |
| Male | 772 (55.4) | 40 (59.7) |  |
| **BMI, median [Q1, Q3]** | 23.4 [21.3, 25.4] | 24.2 [21.6, 25.8] | 0.535 |
| **BMI categories, n (%)** |  |  | 0.623 |
| <18.5 | 64 (4.7) | 1 (3.2) |  |
| 18.5-24.9 | 893 (66.1) | 18 (58.1) |  |
| 25.0-29.9 | 297 (22.0) | 10 (32.3) |  |
| ≥30.0 | 96 (7.1) | 2 (6.5) |  |
| **Residence, n (%)** |  |  | 0.656 |
| Urban | 1,056 (78.0) | 25 (83.3) |  |
| Rural | 297 (22.0) | 5 (16.7) |  |
| **Education, n (%)** |  |  | 0.371 |
| Primary or below | 216 (16.0) | 3 (9.7) |  |
| Lower secondary | 359 (26.5) | 6 (19.4) |  |
| Upper secondary | 310 (22.9) | 10 (32.3) |  |
| Associate degree | 240 (17.7) | 4 (12.9) |  |
| Bachelor's or higher | 228 (16.9) | 8 (25.8) |  |
| **Occupation, n (%)** |  |  | 0.396 |
| Student | 46 (3.4) | 0 (0.0) |  |
| Employed | 437 (32.2) | 6 (19.4) |  |
| Unemployed | 321 (23.7) | 10 (32.3) |  |
| Retired | 71 (5.2) | 2 (6.5) |  |
| Other | 481 (35.5) | 13 (41.9) |  |
| **Lifestyle, n (%)** |  |  |  |
| Smoking, n (%) | 161 (12.4) | 5 (31.3) | 0.041 |
| Alcohol, n (%) | 90 (6.9) | 3 (18.8) | 0.097 |
| **Atopy & sensitivities, n (%)** |  |  |  |
| Elevated total serum IgE | 245 (17.6) | 0 (NA) | — |
| Peripheral blood eosinophilia | 165 (11.8) | 0 (NA) | — |
| Allergen-specific IgE (≥ class 2) | 65 (4.7) | 0 (NA) | — |
| Immediate hypersensitivity reactions | 31 (2.2) | 0 (NA) | — |
| Food allergy | 31 (2.3) | 0 (0.0) | >0.999 |
| Drug allergy | 19 (1.4) | 1 (8.3) | 0.164 |
| Family history of atopic diseases | 137 (9.8) | 1 (25.0) | 0.340 |
| **Comorbidities, n (%)** |  |  |  |
| Asthma | 35 (2.6) | 1 (8.3) | 0.278 |
| Allergic rhinitis | 158 (11.8) | 1 (8.3) | >0.999 |
| Allergic conjunctivitis | 5 (0.4) | 0 (0.0) | >0.999 |
| Chronic urticaria | 50 (3.7) | 2 (16.7) | 0.075 |
| Ichthyosis vulgaris | 6 (0.4) | 0 (0.0) | >0.999 |
| Hypertension | 93 (6.9) | 1 (8.3) | 0.581 |
| Coronary heart disease | 16 (1.2) | 0 (0.0) | >0.999 |
| Type 2 diabetes mellitus | 42 (3.1) | 2 (16.7) | 0.056 |
| Psychiatric disorders | 12 (0.9) | 1 (8.3) | 0.110 |
| Data are presented as median [IQR] for continuous variables and n (%) for categorical variables. P values were calculated using the Wilcoxon rank sum test for continuous variables and Pearson chi-square tests or Fisher exact tests for categorical variables as appropriate. Note: Total serum IgE missing indicates that total serum IgE status was not recorded at enrollment in the site-entered database. Abbreviations: PN, prurigo nodularis; IgE, immunoglobulin E; BMI, body mass index; IQR, interquartile range. | | | |
