# Supplementary material for: IgE-defined endotypes reveal distinct clinical profiles of prurigo nodularis compared with atopic dermatitis: a multicenter study in China
Source: Front Allergy. 2026 Feb 25;7:1769768. doi: 10.3389/falgy.2026.1769768 (PMC12975732; doi:10.3389/falgy.2026.1769768)
Supplement: Supplementary Table S5 — Model performance and internal validation. [file Table5.docx]

**Table S5. Model Performance and Internal Validation**

| Comparison | Apparent AUC (imp-mean ± SD) | DeLong AUC imp1 (95% CI) | Optimism | Optimism-corrected AUC | Bootstrap test AUC (95% CI)^a^ | Brier | Tjur R² | MI-stacked Intercept (95% CI) | MI-stacked Slope (95% CI) |
| --- | --- | --- | --- | --- | --- | --- | --- | --- | --- |
| AD vs PN | 0.730  (0.004) | 0.733(0.714-0.752) | 0.017 | 0.713 | 0.721  (0.720-0.723) | 0.206 | 0.163 | 0.00  (-0.19-0.19) | 1.00  (0.98-1.24) |
| IgEnormalPN  vs  IgEhighPN | 0.737 (0.005) | 0.737 (0.702–0.773) | 0.048 | 0.689 | 0.712 (0.708–0.716) | 0.122 | 0.157 | 0.00 (-0.25–0.25) | 1.00 (0.85–1.15) |
| AD  vs  IgEhighPN | 0.714 (0.007) | 0.720 (0.685–0.754) | 0.047 | 0.663 | 0.690 (0.686–0.693) | 0.130 | 0.102 | 0.00 (-0.29–0.29) | 1.00 (0.82–1.18) |
| AD  vs IgEnormalPN | 0.749 (0.001) | 0.750 (0.730–0.769) | 0.017 | 0.732 | 0.742 (0.741–0.743) | 0.201 | 0.194 | 0.00 (-0.09–0.09) | 1.00 (0.89–1.11) |
| Notes: (a) Discrimination was quantified by the area under the receiver operating characteristic curve (AUC) and corrected for optimism via bootstrap resampling with 1,000 replicates. (b) Calibration was assessed by the calibration intercept and slope; lower Brier scores indicate better overall accuracy. (c)Tjur’s R² reflects the difference in average predicted probabilities between outcome groups. (d) Estimates were pooled across imputed datasets (m = 20) using Rubin’s rules. Abbreviations: PN, prurigo nodularis; AD, atopic dermatitis; IgE, immunoglobulin E; AUC, area under the ROC curve. | | | | | | | | | |
